# Start small and let it build; a mixed-method evaluation of a school-based physical activity program, Kilometre Club

**DOI:** 10.1186/s12889-022-14927-7

**Published:** 2023-01-19

**Authors:** Teisha Schirmer, Andrew Bailey, Nicola Kerr, Alison Walton, Linda Ferrington, Michael E. Cecilio

**Affiliations:** 1 Mid North Coast Local Health District, Coffs Harbour, NSW Australia; 2 Rural Clinical Campus, University of New South Wales, Port Macquarie, NSW Australia; 3 New South Wales Ministry of Health, St Leonards, NSW Australia

**Keywords:** Physical activity, Primary school, Health promotion, Program evaluation, Implementation science

## Abstract

**Background:**

Despite the benefits of physical activity, there is minimal research focusing on factors that influence real-world school-based physical activity programs. Kilometre (KM) Club is an Australian grassroots program which aims to increase physical activity in students through the completion of an outside walk or run at school. This small-scale pilot evaluation aimed to examine families, teachers and principals’ perceptions of the benefits, enablers and barriers of KM Club. It also aimed to examine the effects of KM Club on student’s physical activity levels during the school day.

**Methods:**

Four regional New South Wales (NSW) primary schools participated in this study. 26 families, four teachers, and two principals from School A, C, B and D completed semi-structured interviews to understand their perceptions of KM Club. 21 students completed emotional state-scales to understand their emotions when participating in KM Club. 141 students from Schools B, C and D participated in step-count measures using accelerometers.

**Results:**

Families, teachers and principals reported a range of benefits such as improved social connectedness, wellbeing, home and classroom behaviours, participation in sport and fitness levels. Enablers consisted of champion engagement, incentives, versatile facilities and integration with other school activities. Identified barriers included the weather and environment, program timing and health issues. Most students reported that participating in KM Club made them feel proud, confident and fantastic. School B reported a significant increase in students' daily step counts on KM Club days compared to non-KM Club days (+ 15%; *p* = 0.001), while School C reported no significant changes (-5%; *p* = 0.26). School D reported a significant increase in the number of daily steps taken by KM Club participants compared with non-KM club participants (+ 10%; *p* = 0.024).

**Conclusion:**

There is no one-size-fits-all approach to implementing school-based physical activity initiatives. However, it appears that flexible and adaptable factors are important to the successful implementation of school-based programs, such as KM Club. This study revealed a variety of self-reported health, wellbeing and educational benefits for students, as well as an increase in student’s physical activity levels at 2 of the 3 schools participating in the quantitative data collection. This pilot evaluation may help to inform future design, implementation and scale-up of KM Club and school-based health promotion programs, potentially improving child health, wellbeing and educational outcomes.

**Trial registration:**

(LNR223 – LNR/19/NCC/45).

**Supplementary Information:**

The online version contains supplementary material available at 10.1186/s12889-022-14927-7.

## Background

Regular physical activity has many physical, mental and psychosocial health benefits for children and reduces the risk of chronic conditions later in life [1–4]. Despite the reported benefits of physical activity, only 23% of Australian children aged 5–14 years meet the recommended 60 min of physical activity per day [5]. A recent scoping review demonstrated a further decline in physical activity and incline in sedentary behaviours of children and adolescents internationally (including Australia) since COVID-19 [6]. Considering this, there is an urgent need for the implementation of acceptable, feasible and effective interventions which increase children’s physical activity.

Schools are important settings for the implementation of health promotion programs that promote physical activity [7]. In Scotland (UK) many schools run a physical activity program called the Daily Mile. The Daily Mile has exhibited multiple health benefits including improved concentration, reduced sedentary behaviour, increased fitness and improved BMI (Body Mass Index) and waist-circumference [8]. Qualitative studies have also shown that simple core factors including supportive organisational environments, incentives and flexible delivery models influence successful implementation of the Daily Mile [9, 10]. Although these studies provide valuable insight into implementing school-based physical activity programs, it is important to examine the perspectives from a wide range of implementers and participants across several schools, within an Australian context.

In Australia, multiple schools have initiated a similar program called the Kilometre Club (KM Club). KM Club is a school-based physical activity program that provides children with the opportunity to run walk or jog on an outside course at a designated time of day. It is a concept that schools adopt and adapt into their school culture. This grassroots program was initiated in 2014 by a deputy principal in a large regional primary school (613 students across Kindergarten to Year 6) in Port Macquarie, New South Wales (NSW). KM Club was introduced to improve student fitness but may also have positive impacts on health and wellbeing. Since its establishment, student participation has grown exponentially and the program has expanded into multiple primary schools across the Mid North Coast (MNC) in NSW. The delivery model varies across schools, as the program has been adapted to each school’s context, for example, before-school vs during-school programming.

To date, there is no evidence to demonstrate the effectiveness of the KM Club or the factors associated with its implementation. Systematic evidence shows the benefits of school-based physical activity programs [11–13], yet few studies have evaluated the implementation factors of grassroots programs. To bridge the gap in evidence between design, piloting, adoption and scale-up of effective school-based physical activity programs, it is important to understand the factors that influence program implementation [14, 15]. KM Club has evolved from a “bottom up” approach, which contrasts with many well-funded, randomised control trial population health school-based programs. Therefore, existing programs developed under complex, real-life circumstances (like KM Club) should be evaluated.

This small-scale pilot evaluation aimed to examine the benefits, enablers and barriers of KM Club from the perspectives of teachers, principals and families. It also examined the effects of KM Club on student’s physical activity levels during the school day. Due to the pragmatic real-world nature of this program evaluation and the limitations to comprehensiveness and consistency of data captured from participating schools, we were unable to be conclusive about the KM Club’s effects on students’ physical activity. However, the information we did glean provides us with a more progressive understanding of the program’s acceptability, potential effectiveness and the factors influencing its implementation. The findings from this pilot evaluation may therefore provide evidence to support a larger-scale evaluation and up-scale of KM Club.

## Methods

### Study design and ethics

This study used a mixed-methods design to address the research aims and gain a deeper understanding of the KM Club. All participants provided informed consent and ethical approval was obtained from the Executive Committee of the North Coast Human Research Ethics Committee (HREC) (LNR223 – LNR/19/NCC/45). The NSW Ministry of Health provided funding for commissioned research agency, Cultural and Indigenous Research Centre Australia (CIRCA), to undertake the qualitative component of this research project in collaboration with Mid North Coast Local Health District (MNCLHD) Health Promotion.

### Settings and participants

Four MNC primary schools who implement KM Club participated in this study. These schools are referred to as School A, B, C and D for de-identification purposes. At School A, KM Club is implemented before-school by multiple parents. School B, also implemented KM Club before-school by one teacher. At School C, KM Club is implemented before-school by one principal. At School D, KM Club is implemented during-school time by multiple teachers.

To respect individual schools needs and ongoing relationships, schools determined the data sources that were able to be collected from their school. Overall, 26 families (students and their parents/carers), 4 teachers and 2 principals participated in qualitative data collection from Schools A, B, C and D. 141 students (Kindergarten to Year 6) from School B, C and D participated in quantitative data collection. School A opted to only provide qualitative data and therefore no quantitative data was collected. Table 1 provides a breakdown of what data was collected from each school.

**Table 1:** Data collected from each school

School	Delivery model	Champion (implementer)	Qualitative Data Collected	Quantitative Data Collected
School A	Before-school	Multiple parents	Interviews with 1 principal and 7 families Emotional state-scales with 5 students	No quantitative data was collected from School A
School B	Before-school	One principal	Interviews with 1 teacher, 1 principal and 7 families Emotional state-scales with 7 students	Step counts from 41 students on KM Club Days and Non-KM Club Days
School C	Before-school	One teacher	Interviews with 1 teacher and 7 families Emotional state-scales with 2 students	Step counts from 29 students on KM Club Days and non-KM Club Days
School D	During-school	Multiple teachers	Interviews with 3 teachers and 6 families Emotional state-scales with 7 students	Step counts from 47 KM Club participants and 24 non-KM Club participants

### Recruitment

The MNCLHD Health Promotion department engaged the four KM Club schools in June 2019. Following school recruitment, principals from each school identified key teachers and/or principals who had played a significant role in the implementation of KM Club. Teachers and principals were invited to participate in qualitative interviews and to provide assistance with recruiting parents/carers and students in both the qualitative and quantitative measures. A researcher from CIRCA then phoned each parent/carer to invite them to participate in the qualitative interviews, inviting families of students who do and do not participate in KM Club. KM Club participants and non-KM Club participants were invited to participate in the quantitative measures. However, School B and C opted to only recruit KM Club participants to prevent disruption to non-participants' schedules. Families were required to complete a Participant Consent form for both qualitative and quantitative measures.

### Qualitative measures

To increase rigour and reduce bias a researcher from CIRCA conducted semi-structured interviews with families, teachers and principals. Family interview questions focussed on perceived enablers, barriers, benefits and disadvantages of participating in KM Club, and any program improvement recommendations. Teachers and principal’s interview questions focussed on factors influencing successful implementation and sustainability, perceived benefits and disadvantages, and recommendations for schools in their establishment of KM Club. The interview schedule from The Daily Mile study informed the interview questions in this study [9].

Students participating in the interviews completed paper-based emotional state-scales (a self-rating against coded emoji’s) to understand and capture their feelings about the program. Students could choose multiple responses. Simple visual and interactive tools are useful in engaging children in research as it gives them an opportunity to provide responses without pressure to identify the “correct” answer [16].

### Thematic analysis

In this study, qualitative data was analysed using an interpretive approach to thematic analysis [17]. Thematic analysis is flexible and can be adapted to different research participants and settings [17]. This methodology can also be useful for examining the perspectives of different research participants and can outline similarities and differences between groups [17]. To increase rigour and reduce bias, two researchers from CIRCA led the qualitative thematic analysis. Firstly, the digitally recorded interviews were transcribed and read to ensure that the data had been accurately transcribed. Once the researchers were familiar with the data, transcripts were independently labelled and coded to identify overarching themes. During the initial analysis, the researchers from CIRCA discussed the developed themes and identified areas lacking exclusivity and in need of further investigation. A second analysis was independently conducted by co-authors within MNCLHD for local contextualisation, quality control and to help further refine themes. Regular discussion, comparison and reflexive consideration of the data occurred among the researchers to ensure the coded data adequately reflected themes. Themes were re-examined to ensure there were no new emerging themes, thematic saturation was achieved and that the themes answered the research questions. Lastly, the researchers identified quotes and comments from the transcripts that reflected the themes. Data analysis was facilitated by NVivo V.10 (QSR International Pty Ltd) software.

### Quantitative measures

Students’ physical activity outcomes were measured using accelerometers (Actigraph GT3X + and GT3X models). Accelerometers are identified as a valid, reliable and objective method of measuring child and adolescent physical activity outcomes [18–20]. Due to the prohibitive costs for Moderate to Vigorous Physical Activity (MVPA) data analysis, only step counts were extracted. Mean steps during the school day was the primary outcome of the quantitative data. Study participants wore their accelerometers from 8am Monday until 3pm on Friday for 2 rounds of data collection during the school year to account for variations in school programming. Teachers kept manual participation records as a way of triangulating the accelerometer data collection.

### Statistical analysis

Quantitative analysis was conducted within the study team using SAS V.9.2 (SAS Institute Inc, Cary, North Carolina, USA). Linear mixed models, with a child level random effect for repeated measures on student’s, were used to investigate the effect of KM Club on step counts during the school day. A subgroup analysis on mean step counts by gender was also performed for each school. Data was collected over two rounds (one week in Term 3 and one week in Term 4). Significance thresholds were set at 0.05. The predictor of interest varied for each school due to the heterogeneity between each school’s delivery model and absence of non-participant data at School B and C. The predictor of interest for Schools B and C was KM Club days (KM Club day’s vs non–KM Club days, among the same students) as School B and C opted to only recruit KM Club participants to prevent disruption to non-participants' schedules. The predictor of interest for School D was KM Club participants (KM Club participants’ vs non-KM Club participants).

### Qualitative findings

#### Sample characteristics

Twenty-six families were interviewed across four school locations, as follows: School A (*n* = 7), School B (*n* = 7), School C (*n* = 6) and School D (*n* = 6). From the 26 families, interviews were conducted with 11 parents/carers and their children who had participated in KM Club; 5 parents/carers and their children who had not participated in KM Club; and 10 parents/carers and their children where at least one of their children had participated in KM Club, but the other child(ren) had not participated.. Four teachers and two principals were also interviewed in total. Interviews lasted approximately 60 min, with 24 interviews conducted face-to-face and two conducted by telephone.

### Establishment and delivery models

KM Club was established in a large regional primary school (613 students) in 2014 by the deputy principal (School A). The popularity of the program and its perceived benefits saw an organic growth in parent engagement, supervision and participation. As a result, parents are now the driving force behind KM Club at School A. In 2016, KM Club was introduced to two small regional schools at School B (250 students) and School C (231 students) by teachers who had been previously exposed to the program. These teachers established the program to improve student fitness and classroom behaviours and prepare them for cross-country and sports carnivals. Students at Schools A, B and C can choose to participate in KM Club as a before-school program. School D established KM Club in 2019 (312 students) by a teacher who was passionate about increasing children’s physical activity to improve health and wellbeing outcomes. Similar to the Daily Mile, School D implements the program during-school hours to enable participation for all students. Most teachers at School D choose to run the program after recess, in class hours. As KM Club is run during-school hours at School D, only students participate if their teacher elect for their class to join. Parents do not participate at School D. Further characteristics of each school’s delivery model is detailed in Table 2.

**Table 2:** KM Club delivery models at each school

School	Model	Days per week	Duration	Weeks	Terms	Participants	Champion (implementer)	Location run
School A	Before-school	5	30 min	1–6	2 and 3	Students, families and teachers	Parents	School rainforest and school oval
School B	Before-school	4	30 min	1–5	1, 2 and 3	Students and occasionally parents	1 teacher	School oval and cross-country track
School C	Before-school	2	30 min	1–10	1–4	Students and occasionally parents	1 principal	School oval and cross-country track
School D	During-school (in class)	5	15 min	1–10	1–4	Students and teachers	Multiple teachers	School oval

### Perceived benefits

#### Social connection

KM Club has provided children and parents/carers the opportunity to socialise and make new friends. One child commented, *“It’s really good exercise for the morning and I like hanging out with my friends.”* One parent who participates in KM Club stated, *“It’s a social thing for me too, because I’m down there chatting with the other mums.”* KM Club has also helped facilitate more exercise within families, encouraged family bonding and improved their connection with the school community. *“My husband really enjoys being able to do something with the kids at school that’s active and something that he feels he can be involved in.”* (Parent). Another parent commented, *“It’s nice to run around with the kids and have that bit of time and see the kid’s friends.”*

#### Wellbeing

Some parents and teachers have perceived reductions in anxiety among children who participate in KM Club. One teacher reported that KM Club might contribute to improved behaviours before and during class for students: *“They love participating and they're finding that that alleviates their anxiety for school which flows onto their academic work.”* One grandparent also perceived that their grandchild’s anxiety regarding school attendance has reduced since commencing KM Club, stating: *“The day KM Club started he was just a completely different child. He got out of the car, couldn’t get here quick enough, had to be here early and raring to go. I haven’t had any problems with him since… KM Club was his saviour and he’s come to school every morning, even when it finished.”*

#### Home and classroom behaviour

Some teachers expressed an improvement in students learning readiness and classroom behaviours as the greatest benefits of the program. One teacher stated, *“I’ve noticed confidence and improved behaviour.”* Some teachers recognized improved concentration, with one teacher stating, “*Students are more settled and ready to learn.”* Positive impacts on behaviour has led School C to implement short and regular physical activity breaks into classroom lessons. Parents/carers reported improved behaviours at home, including an increase in readiness for school in the mornings and improved compliancy regarding routines in the home. One parent expressed *“KM Club helps them get organised in the morning… they’re so keen to get to school.”*

#### Fitness and participation in physical activity

Many parents/carers, teachers, principals and students reported that KM Club has improved children’s fitness and encourages greater participation in sport, with one child stating: *“I like that you get to stay fit and healthy.” “Fitness benefits for children have resulted in greater participation in events at the athletics carnival.”* (Principal). One parent commented on the benefits for children who are typically disinterested in competitive sports: *“It’s a solo kind of activity that they can challenge themselves rather than being challenged against someone else.”*

### Enablers and barriers

#### Champion engagement

Active engagement and commitment of champions was perceived as vital to the establishment, implementation and sustainability of KM Club. At each school, champions consisted of either principals, teachers and/or parents/carers. The deputy principal at School A, who was responsible for establishing KM Club stated, *“Start small and let it build”*. Champions believe in the program and the positive effects it can have on their school community, with one stating, *“One teacher who is keen to do something, that's all it took.”* KM Club champions serve as role models encouraging participation through positive attitudes towards the program. Champions organise and maintain the course and create incentives to encourage participation. School A had a very active parent base which assisted with most of the maintenance and sustainability of KM Club. One teacher stated, *“We definitely would not be able to do this program without the help of our parents.”*

#### Incentives and motives

Incentives have been implemented fluidly to encourage participation, varying not only by school, but also by class. To encourage and assist children in setting and achieving personal goals, champions monitored student participation by recording the number of laps or total distance completed on stamp cards. One school also rewarded students with certificates (bronze, silver, gold and marathon) based on their total amount of kilometres completed in one week. One parent noted, *“He’s motivated by the certificates… he just wants to get as many done as he can.”*

One teacher identified motivation maintenance as a barrier, *“Maintaining their enthusiasm for it is probably the hardest thing.”* However, to increase participation and maintain motivation, some teachers run with their students, provide verbal encouragement, play music through a speaker and encourage students to kick or throw a ball. One school uses Google Maps to track the total distance run around Australia, creating a common goal for the entire school community, and a whole-school approach to increasing physical activity. *“Collectively our school has run all the way around Australia and through to Uluru.”* (Teacher). In the early stage of implementation, School B utilized external support from a supermarket, through the donation of fruit and vegetables which were provided to students to eat after completing KM Club.

While teachers, principals and parents agreed that incentives were vital for encouraging participation in the early stages of implementation, they felt they were not required for long-term sustainability *“We haven’t found participants have needed any extra motivation to do it. Less active kids, perhaps they’re in need of a little verbal encouragement.”* (Teacher).

#### Adaptable and flexible delivery models

Each school’s delivery model varies, as they have adapted KM Club to fit their school’s context. Although KM Club follows a generic procedure for implementation, champions have autonomy over when and how they execute it. KM Club caters to all fitness levels and has provided participants with flexibility to run, jog, walk, kick or throw a ball at their own pace, for as long as they like. *“Some will come and walk but others are determined to do as many laps as they can… so long as they’re moving… it’s better than sitting, waiting for the bell to go”* (Teacher).

##### Before-school model

The before-school model reduces the commitment required by teachers, allowing the program to run for longer than the during-school model, without interfering with curriculum commitments. One teacher stated, *“This is something that students can do without costing key learning time… it’s easy to run, it’s no extra commitment really from staff.”* The before-school model also enhanced a whole-of-school approach to physical activity. Some parents expressed that this model encouraged parent participation as it doesn’t interfere with busy work schedules. However, the before-school model can present barriers for children who are unable to arrive in time for the program. One parent commented: *“It can be hard if you’re [*child’s*] on a late bus or if you are a parent that can’t get your child to school early enough.”*

##### During-school model

There were some differences in the frequency and level of engagement at School D. The teacher and principal at School D believed KM Club required minimal time requirements and costs, while limiting disruption to classroom learning. *“It’s very low resource, it’s very low time consuming. Other than that, 15 min, that’s it.”* (Teacher). However, it was expressed that some teachers stopped implementing it as they felt it interfered with learning time. The teacher and principal at School D felt the during-school model enabled teacher autonomy by allowing the flexibility to timetable KM Club to suit their daily plan or children’s behavioural needs. It also provided all students (at the teacher’s discretion) with the opportunity to be physically active, contributing to the NSW Department of Education 150 minutes of mandatory physical activity.

#### Integration with other school activities

Teachers recognized that integrating KM Club in other school activities has benefits for participation, champion engagement, executive sponsorship, and sustainability and ensures the program is integrated in whole-of-school planning. KM Club aligns with elements of the school curriculum *“It’s really good for talking about maths and numbers and setting goals.”* (Teacher). Schools also linked KM Club to external sporting events (e.g., cross-country and athletics carnivals) to improve student’s fitness levels and confidence to participate, with notable increases in representation in sporting events. *“It improves the children’s fitness enormously leading up to those carnivals.”* (Teacher).

#### Weather

Multiple champions and families identified weather as a barrier to participation, specifically warm weather and wet weather. Despite this, most teachers and principals expressed that they would like to implement KM Club in all weather and seasons but are limited by the hot summer months, unsafe surfaces and inappropriate footwear. Teachers and parents expressed that the heat can be challenging and can lead to exhaustion and hygiene concerns, such as body odour. One teacher noted, *“I was doing it in term 1 and term 4 but then they’re running in the heat… numbers were dropping off … it was just too hot.”* School C and D have overcome this barrier by encouraging students to walk in the warmer months. *“During the warmer month’s majority of the students walk to reduce the impact of the heat on hot KM Club days.”* (Teacher). Some teachers also expressed a lack of wet weather options can hinder program implementation; *“Some days if it has been wet, we can’t go because their shoes will be atrocious.”* (Teacher).

#### Facilities

All of the schools involved in the study have access to large greenspaces, allowing for variation in tracks. Teachers identified that KM Club can be adapted to suit a variety of school environments, in varying locations, with different facilities. Teachers and principals emphasized the importance of being innovative when creating the track, ensuring adaptable tracks. “*I have seen a similar program run at another school* [in the city] *on a block around the footpath of the school…I guess it’s just thinking creatively to find a track and how you can make it work.”* (Teacher). One parent reported on their child’s disengagement, *“He’s not bored of KM Club, just the track.”* Some schools vary the track on a term-by-term or yearly basis to maintain student interest and ensure all students abilities are catered for. *“We’ve got a short track and long track… it caters for all their abilities and sometimes they want to do one of each and sometimes they want to go forwards or backwards.”* (Teacher). However, one teacher who previously worked in a metropolitan school expressed that it can be challenging to maintain student interest when green spaces and facilities are limited: *“It’s a lot harder because of smaller playgrounds… it was concrete… what can you do with that?”.*

#### Health issues

Some children struggle to participate for health reasons, particularly asthma, disability or special needs, though this varies depending on the child, specific condition and activitities undertaken. Some children with asthma experience increased exhaustion, which affected their school work. *“My child is an asthmatic, she has done a couple of rounds but finds that she gets quite weak afterwards… it interrupts a lot of the actual school work for her if she was to go and run.”* (Parent). Another parent expressed, *“My son has autism so he’s quite stuck in his ways. I think that if the whole class did it then it would be less of a drama for him.”* However, the program can be adapted to support student’s needs. One child with asthma expressed that having the flexibility to walk has enabled participation, stating *“If you didn’t bring your puffer, you are allowed to just walk.”*

### Emotional state-scale

Twenty-one emotional state-scales were collected from interviewed students (see Table 3)*.* Most reported that participating in KM Club made them feel proud, confident and fantastic. Of the 81 responses selected, 75 were identified as positive emotions; four responses were negative and two were neutral.

**Table 3:** Feeling states among interviewed children who participated in KM Club

Emotion Rating	School A	School B	School C	School D	Total
Proud	5	2	6	7	20
Confident	5	—	4	6	15
Fantastic	5	2	4	4	15
Fit	3	2	5	6	13
Strong	3	1	4	4	12
Shy/Embarrassed	—	—	1	1	2
Bored/Nothing	1	—	1	—	2
Weak	—	—	1	—	1
Unfit	—	—	1	—	1
Awful	—	—	—	—	0

## Quantitative results

### Sample characteristics

Three of four schools took part in the quantitative data collection. Across all three participating sites, 141 children were included in round 1 data collection (Term 3, September 2019). Fifteen children were lost to follow up, resulting in 126 participants in round 2 data collection (Term 4, October 2019). Table 4 illustrates the study participant characteristics in each school and the total participants in each age group. Across all schools, the mean age of participants was 10.4 years.

**Table 4:** KM club sample characteristics

**Age (years)**	School B (*n* = 41)	School C (*n* = 29)	School D KM Club participants (*n* = 47)	School D KM Club non-participants (*n* = 24)	Total
5–6	4	2	0	0	6
7–8	3	4	0	0	7
9–10	14	12	27	1	54
11–12	19	11	28	4	62
13–14	1	0	4	7	12

### Step count data

Step count data was collected in three of the four schools (B, C and D) as School A opted not to participate in step count data collection. Students step counts were collected over 2 school weeks (10 days) at each school participating in the quantitative data collection. KM Club ran 4 days per week at School B, 2 days per week at School C and 5 days per week at School D. Table 5 presents a comparison between School B student's step counts on their KM Club days (up to 4 days per week) with their non-KM Club days (at least 1 day per week). Linear mixed-models' analysis revealed a statistically significant difference between the groups overall, whereby the number of daily steps taken on KM Club days was significantly higher than non-KM Club days (+ 15%; *p* = 0.001). Table 5 also presents a comparison between the mean daily step counts of School C student' on their KM Club days (up to 2 days per week) with their non-KM Club days (up to 3 days per week). There were no significant differences between student’s total KM Club days and their non-KM Club days.

**Table 5:** School B and C mean daily step counts

School	Students mean daily step counts on KM Club days	Students mean daily step counts on non-KM Club days	Difference between treatment groups (95% CI)	*P* value
School B (*n* = 41)	8944.92 (2003.20^a^)	7594.67 (2066.05^a^)	1251.01 [672.27; 1829.75]	< 0.001*
School C (*n* = 29)	7665.83 (1279.59^a^)	8057.34 (1966.61^a^)	-212.12 [-584.37; 160.13]	0.26

^a^ Standard Deviation

^*^ *p* < .05

Table 6 presents a comparison between School D students who participated in KM Club (*n* = 47) and those who did not (*n* = 24). The number of daily steps taken by KM Club participants was significantly higher than that of non-KM club participants (+ 10%; *p* = 0.024). This is a small data set as we were only able to compare participants to non-participants at School D.

**Table 6:** School D mean daily step counts

School	KM Club participants mean daily step count	Non-KM Club participants mean daily step count	Mean difference between treatment groups (95% CI)	*P* value
School D (total *n* = 71)	6120.36 (1643.64^a^)	5559.38 (2003.73^a^)	547.59 [74.27; 1020.91]	0.024*

^*^p < .05

^a^ Standard Deviation

## Discussion

The aim of this small-scale pilot study was to examine the benefits, enablers and barriers associated with the implementation of KM Club in four NSW primary schools, and its effects on physical activity behaviours of children throughout the school day. The findings from this study suggest that having flexible and adaptable factors that meet the school context can influence successful implementation of KM Club. Parents, teachers and principals reported that the program can positively influence health, wellbeing and educational outcomes within a school community. Quantitative data demonstrated a significant improvement in physical activity outcomes in two of the three schools that run the program 4 + days a week.

A delicate balance exists between achieving consistent implementation across multiple contexts, while providing the flexibility for local sites to adapt the intervention [21]. This study has shown how this balance can be achieved by identifying factors that are essential, yet flexible, for the program’s success. Several factors identified in this study are also reflected in the implementation literature, including champion engagement, teacher and student autonomy, and versatile facilities [22, 23].

KM Club's adaptability has potentially contributed to its transferability from inception, however preserving the integrity of key enabling factors is important.

The flexibility in delivery models was fundamental to participants’ perceptions of the program’s value and longevity. This study has demonstrated how flexible and adaptable delivery models can inspire innovative approaches to overcoming barriers to implementation. One school's barriers have been found to be an enabler at another. For example, the before-school model may not be accessible to all children, whereas the during-school model permitted whole-school participation. However, the before-school model encouraged socialization among children, parents, and teachers through a community-based approach.

The reality of implementing health promotion programs in schools requires the active engagement and commitment of a variety of champions in planning and implementation, as well as the adaption of programs to local contexts [24, 25]. Further, multiple studies have demonstrated the importance of individual school members in influencing implementation and sustainability success or failure of any change effort in schools [26–28]. This was evident in KM Club, where teachers, principals and parents played a vital role in implementing, maintaining and adapting the program to meet the school context. This is reflected in the implementation literature, where the grassroots bottom-up approach has consistently led to better implementation and outcomes [29, 30].

Families, teachers and principals perceived health, wellbeing and educational benefits for children who participate in KM Club. Interviewees suggested that KM Club contributed to children’s increase in fitness as it provided an additional opportunity for children to participate in physical activity. Most parents and teachers recognized the benefits of children expending energy through physical movement, contributing to improvements in children’s learning readiness, concentration and classroom and home behaviours. This is supported in recent literature, which shows positive impacts of physical activity on academic achievement [31–33]. However, some teachers at School D were concerned that the during-school model may affect learning time, a finding that was similar in the Daily Mile study [10]. The findings from this study may suggest that time invested in the KM Club can produce additional cognitive, educational and behavioural benefits.

Children’s physical activity levels during school hours vary according to the temperature and season [34]. This was highlighted in this study, where hot temperatures were frequently raised as a barrier to implementation. Similarly, an Australian study has suggested that children’s physical activity levels were lower in summer compared to winter, due to the hotter temperatures [35]. However, some schools have navigated this barrier by walking in the hotter months. This study has demonstrated that having adaptable and flexible models to fit the season can provide student’s with further opportunities to be physically active.

## Strengths and limitations

This is the first study to evaluate KM Club, and one of few studies to evaluate the factors that influence the implementation of grassroots school-based physical activity programs. In this study, mixed-methods were utilized to gain a deeper understanding of the implementation of KM Club as well as perceived health and wellbeing outcomes. This study offers the opportunity to better understand how to support schools to embed physical activity into children’s weekly life and make it easy, fun and accessible.

A limitation of this study is the inconsistency in the predictor of interest due to restrictions on quantitative data collection. The decision to respect individual schools and sustain relationships with schools was important in this study. As a result, the data collected at each site varied. This highlights the complexities of collecting consistent data in school settings, particularly when evaluating existing flexible program deliveries. However, we believe the findings from this small data set provide an indication as to the potential effectiveness of the KM Club on students’ physical activity and can inform a more comprehensive evaluation in future. Other limitations include the inability to account for MVPA outcomes due to prohibitive costs for data analysis and also the weather disruptions when collecting data at School C. Given these limitations, physical activity results should be viewed as preliminary with further research needed.

## Conclusion

There is no one-size-fits all approach to implementing school-based physical activity programs, such as KM Club. However, having implementation factors, which are flexible and adaptable to suit the school context, appear to be key to the successful implementation of KM Club. This study has shown a variety of self-reported health, wellbeing and educational benefits for children, as well as an increase in their physical activity levels. Future research could examine the success of implementing KM Club in new schools based on enablers identified in this study. Future research could also examine the weekly dosage of KM Club required to achieve improvements in physical activity, wellbeing and educational outcomes in a larger sample size of schools. These findings can assist schools, policy makers and health researchers in implementing KM Club and other school-based health promotion interventions, potentially improving child health, wellbeing and educational outcomes.

## Supplementary Information

**Additional file 1.** Interview schedule for families of children who do not participate in KM Club (non-participating).

**Additional file 2.** Interview schedule for families of children who do participate in KM Club (participating).

**Additional file 3.** Interview schedule for teachers and/or principals.

## Data Availability

The datasets generated and analysed during the current study are not publicly available, but are available from the corresponding author on a reasonable request through an E-mail (Teisha.Schirmer@health.nsw.gov.au).
