# Supplementary material for: Start small and let it build; a mixed-method evaluation of a school-based physical activity program, Kilometre Club
Source: BMC Public Health. 2023 Jan 19;23:137. doi: 10.1186/s12889-022-14927-7 (PMC9850327; doi:10.1186/s12889-022-14927-7)
Supplement: Supplementary file 1 — Additional file 1. Interview schedule for families of children who do not participate in KM Club (non-participating). [file 12889_2022_14927_MOESM1_ESM.docx]

**Interview schedule for families (non-participating)**

Firstly, I want to say thank you for taking the time to talk with me about the KM Club. My name is X and I am a X for X. This interview is part of research to understand what makes the KM Club successful. We know that KM Club has been run at your child’s school since *[year]* and while there are some families that participate in KM Club, there are many others that do not for many reasons. We are keen to learn today about some of the reasons why some families decide not to participate in KM Club. Your input is really valuable as it will help other schools to run this initiative successfully.

The discussion will take anywhere between 10 to 30 minutes. My job is to facilitate this discussion and ask some questions to help get us talking. Just a few points before we start:

1. THERE ARE NO RIGHT OR WRONG ANSWERS

- Your individual experiences and opinions are important

*[Face-to-face interview]*

1. WHAT IS SAID IN THIS ROOM STAYS HERE

[*Phone interview*]

WHAT IS SAID ON THE PHONE STAY HERE

- I want you to feel comfortable sharing if sensitive issues come up
- We don't identify anyone by name in our reports and you will remain anonymous

1. WE WILL BE TAPE RECORDING THE DISCUSSION

- As mentioned in the participant information sheet, I would like to record the interview. With your permission, a researcher will type up what we have discussed, combine this with other interviews we are conducting and share the transcript with the Mid North Coast Local Health District. We will then look at these and try and figure out what themes and ideas have come out of the discussions. This data will be used to help other schools plan and deliver the KM Club more effectively.
- All personal identifiers will be removed from the transcript prior to sharing. The transcript will only be used for research purposes.
  - Please confirm that I have your permission to record the interview?
  - Please also confirm you are happy to have the de-identified transcripts shared with the Mid North Coast Local Health District
- [*If the response is ‘Yes’*] Thank you. I will now start the interview.
- [*If the response is ‘No’ to recording*] I am unable to conduct this interview without your permission to record the interview. Thank you for your participation in this research to date. [*End interview*]

*****Turn tape recorder on*****

**Families of non-participating students**

**Introduction**

- **Firstly, have you heard much about the KM Club and if so, what do you think it involves?**
- **Are there any types of physical activities that your family regularly participate in? If so what are they?**

*[If they do participate]*

- **How often does your family participate in these activities?**

1. **Intervention implementation**

- **As I said at the start, while there are some families that participate in KM Club, there are many others that do not. So today we’d appreciate if you could help us to understand some of the reasons your family may not participate. What are some of the challenges which prevent your child from attending KM Club?**

*Prompt: other commitments, time, transport, money*

- **From what you know about Km Club, what don’t you like about it?**

*Prompt: Childs behaviour, parents/child have to wake up earlier, have to bring a change of clothes to school, track is too long, less time for other activities*

- **Are there any other weaknesses about the program that you think would affect other families?**
- **Is there anything you would change and/or improve about the program?**
- **From what you know about KM Club, what do you think are the benefits of KM Club?**

*Prompt: Better behaviour, improved health, fitter, happier, improved attention in the classroom, good social interaction with friends*

[Depending on reason for not participating]

- **Would you like your child to participate in KM Club in the future?**
- **Finally, is there anything else you’d like to add about what we’ve discussed today?**

**End**

- Thank you
- Reiterate what will happen with the data and next steps (data recorded, transcribed, and combined with other interviews we are conducting and with student physical activity outcomes)
- Can I contact you again if I have any more questions
