# Supplementary material for: Start small and let it build; a mixed-method evaluation of a school-based physical activity program, Kilometre Club
Source: BMC Public Health. 2023 Jan 19;23:137. doi: 10.1186/s12889-022-14927-7 (PMC9850327; doi:10.1186/s12889-022-14927-7)
Supplement: Supplementary file 2 — Additional file 2. Interview schedule for families of children who do participate in KM Club (participating). [file 12889_2022_14927_MOESM2_ESM.docx]

**Interview schedule for families (participating)**

Firstly, I want to say thank you for taking the time to talk with me about the KM Club. My name is X and I am a X for X. This interview is part of research to understand what makes the KM Club successful. We would like to know what does and doesn’t work, the benefits and disadvantages, and what advice you would give to other schools trying to start KM Club.

The discussion will take anywhere between 10 to 30 minutes. My job is to facilitate this discussion and ask some questions to help get us talking. Just a few points before we start:

1. THERE ARE NO RIGHT OR WRONG ANSWERS

- Your individual experiences and opinions are important

*[Face-to-face interview]*

1. WHAT IS SAID IN THIS ROOM STAYS HERE

[*Phone interview*]

WHAT IS SAID ON THE PHONE STAY HERE

- I want you to feel comfortable sharing if sensitive issues come up
- We don't identify anyone by name in our reports and you will remain anonymous

1. WE WILL BE TAPE RECORDING THE DISCUSSION

- As mentioned in the participant information sheet, I would like to record the interview. With your permission, a researcher will type up what we have discussed, combine this with other interviews we are conducting and share the transcript with the Mid North Coast Local Health District. We will then look at these and try and figure out what themes and ideas have come out of the discussions. This data will be used to help other schools plan and deliver the KM Club more effectively.
- All personal identifiers will be removed from the transcript prior to sharing. The transcript will only be used for research purposes.
  - Please confirm that I have your permission to record the interview?
  - Please also confirm you are happy to have the de-identified transcripts shared with the Mid North Coast Local Health District
- [*If the response is ‘Yes’*] Thank you. I will now start the interview.
- [*If the response is ‘No’ to recording*] I am unable to conduct this interview without your permission to record the interview. Thank you for your participation in this research to date. [*End interview*]

*****Turn tape recorder on*****

**Parents of participating students**

**Introduction**

**Now I’ll be asking you both a variety of questions about your experiences with KM Club. Please both feel free to add any comments at anytime.**

*Ask parent*

- **What can you tell me about KM Club at your child’s school? How does it work?**

*Prompt:* How far is the distance, what time does it run, how many people on average turn up, what age groups can participate, who coordinates it

*Ask student (child)*

- **Is there anything you would like to add, [child’s name]?**

*Ask parent*

- **What originally made you decide for your child to participate in KM Club?**

1. **Students participation**

*Ask parent*

- **How many days does your child participate on average in the KM Club a week?**

*Ask student*

- **What kinds of activities do you do in the KM Club?**

*Prompt: e.g. walking, jogging, running, hopping*

1. **Parents participation:**

*Ask parent*

- **And what about yourself, do you participate in the KM Club too?**

[*If the response is ‘Yes’*]

- **What type of activities do you usually perform when participating in the KM Club?**

*Prompt: e.g. walking, jogging, running, hopping*

- **On average, how many days do you attend KM Club each week?**

1. **Intervention implementation**

*Ask parent*

- **What are some of the things which influence and motivate [child’s name] to participate in KM Club?**

*Prompt: easy access to school grounds, parents work schedule, motivation*

*Ask parent*

- **What do you believe are the benefits of participating in KM Club?**

*Prompt: better behaviour, improved health, fitter, happier, improved attention in the classroom, good social interaction with friends*

*Ask student*

- **What do you like about the KM Club?**

*Ask parent*

- **Are there any challenges that make it difficult for [child’s name] to attend KM Club? If so, what are those challenges?**

*Prompt: parent dropping kid to school early, getting kids up earlier, no public transport, not a priority for parent, hard to bring a change of clothing to school*

*Ask student*

- **Is there anything that makes it hard for you to participate in KM Club? If so what are those things?**

*Ask student*

- **Is there anything you don’t like about KM Club? If so what don’t you like?**

*Ask parent*

- **Are there any disadvantages to the KM Club? If so what do you think they are?**

*Prompt: Child is tired at school, parents/child have to wake up earlier, have to bring a change of clothes to school, track is too long, less time for other activities*

*Ask parent and student*

- **Is there anything else you’d like to add about what we’ve discussed today?**

*Ask student and parent*

- **Finally, is there anything you would change and/or improve about the KM Club?**

**End**

- Thank you
- Reiterate what will happen with the data and next steps (data recorded, transcribed, and combined with other interviews we are conducting and with student physical activity outcomes)
- Can I contact you again if I have any more questions?
