# Supplementary material for: Start small and let it build; a mixed-method evaluation of a school-based physical activity program, Kilometre Club
Source: BMC Public Health. 2023 Jan 19;23:137. doi: 10.1186/s12889-022-14927-7 (PMC9850327; doi:10.1186/s12889-022-14927-7)
Supplement: Supplementary file 3 — Additional file 3. Interview schedule for teachers and/or principals. [file 12889_2022_14927_MOESM3_ESM.docx]

**Interview schedule for teachers and/or principals**

**Page 2: Original Teacher/Principal who organised the PA initiative**

**Page 4: Other teachers involved in implementing the PA initiative**

Firstly, I want to say thank you for taking the time to talk with me about the KM Club. My name is X and I am a X for X. This interview is part of an evaluation study focussing on factors which influence successful implementation and sustainability of the KM Club. We would also like to know what did and didn’t work, the benefits and disadvantages, and what advice you would give to other schools trying to start KM Club.

The discussion will take anywhere between 10 to 30 minutes. My job is to facilitate this discussion and ask some questions to help get us talking. Just a few points before we start:

1. THERE ARE NO RIGHT OR WRONG ANSWERS

- Your individual experiences and opinions are important

*[Face-to-face interview]*

1. WHAT IS SAID IN THIS ROOM STAYS HERE

[*Phone interview*]

WHAT IS SAID ON THE PHONE STAY HERE

- I want you to feel comfortable sharing if sensitive issues come up
- We don't identify anyone by name in our reports and you will remain anonymous

1. WE WILL BE TAPE RECORDING THE DISCUSSION

- As mentioned in the participant information sheet, I would like to record the interview. With your permission, a researcher will type up what we have discussed, combine this with other interviews we are conducting and share the transcript with the Mid North Coast Local Health District. We will then look at these and try and figure out what themes and ideas have come out of the discussions. This data will be used to help other schools plan and deliver the KM Club more effectively.
- All personal identifiers will be removed from the transcript prior to sharing. The transcript will only be used for research purposes.
  - Please confirm that I have your permission to record the interview?
  - Please also confirm you are happy to have the de-identified transcripts shared with the Mid North Coast Local Health District
- [*If the response is ‘Yes’*] Thank you. I will now start the interview.
- [*If the response is ‘No’ to recording*] I am unable to conduct this interview without your permission to record the interview. Thank you for your participation in this research to date. [*End interview*]

*****Turn tape recorder on*****

**Original teacher/principal**

(Check whether they still work at the school)

(Determine what physical activity initiative their school implements [km club, running club etc] before interview)

1. **Background information about teacher**

- **How long have you been teaching at [*school name*] for?**

1. **Intervention development, trialling and roll out:**

- **When and how did KM club start in your school?**

*Prompt: Whose idea was it, when did you try and start it, what steps did you take, were parents and children involved, did you try and disseminate the idea to teachers, did you promote the project, did you get external support, was it phase in or all at once?*

- **Why did you decide to try and start the KM Club?**
- **Did you encounter any challenges trying to set up KM Club?**

*Prompt: Motivation (staff, kids), parent’s approval, access to facilities, weather, clothing, risk assessments, space, timetabling?*

- **What helped the most in setting up KM Club?**

*Prompts: External support, resources?*

- **Can you tell me how KM Club works at your school?**

*Prompt: What* ***type*** *of activity is performed during the KM Club? (walking, running); What* ***time, duration*** *and* ***how many days a week*** *is KM Club run at your school?;* ***Where*** *is the track located and* ***how many kilometres*** *is the track?* *Who decides* *when it is used?;* ***Who*** *can* ***participate?*** *(students, parents, teachers);* ***Who*** *runs the program (teachers or parents)?*

- **Has it changed over time – if so how?**

*Prompt: What happened when the field changed?*

1. **Benefits, disadvantages and recommendations**

*[Ask all teachers]*

- **What do you believe are the benefits of KM Club?**

*Prompt: behaviour, health, engagement, attention?*

- **Do you believe there are any disadvantages to KM Club? If so, what are they?**

*Prompt:*  *Kids left out, supervision, tired in class*

- **Do you believe there are any disadvantages to KM Club? If so, what are they?**

*Prompt:*  *Kids left out, supervision, tired in class*

- **Based on what you’ve seen and heard from families, what are some of the things that prevent families from participating in KM Club?**
- **Is there anything else you would like to add in regards to KM Club implementation?**
- **Finally, what top tips would you give to another school looking to start KM Club?**

**End**

- Thank you
- Reiterate what will happen with the data and next steps (data recorded, transcribed, and combined with other interviews we are conducting and with student physical activity outcomes)
- Can I contact you again if I have any more questions?

**Other teachers involved in implementation**

(Determine what physical activity initiative their school implements [km club, running club etc] before interview)

1. **Introduction to teacher and KM Club**

*[Ask all teachers]*

- **How long have you been teaching at [*school name*] for?**
- **Were you teaching when KM Club was originally implemented**?

*[Only ask teachers who were there from the beginning]*

- **When and how did KM club start in your school?**

*[Ask all teachers]*

- **Can you tell me how KM Club works at your school?**

*Prompt: What* ***type*** *of activity is performed during the KM Club? (walking, running); What* ***time, duration*** *and* ***how many days a week*** *is KM Club run at your school?;* ***Where*** *is the track located and* ***how many kilometres*** *is the track?* *Who decides* *when it is used?;* ***Who*** *can* ***participate?*** *(students, parents, teachers)* ***Who*** *runs the program (teachers or parents?)*

- **Has it changed since you first started – if so how?**

*Prompt: What happened when the field changed?*

1. **Teachers role/responsibility**

*[Ask all teachers]*

- **What is your role within KM Club?**

*Prompt: do you just supervise? Or do you participate too?*

- **How often do you supervise KM Club?**
- **What motivates you to coordinate the KM Club?**
- **Do you receive any support regarding your commitment?**

1. **Intervention development and implementation**

*[Only ask teachers who were there from the beginning]*

- **Did you encounter any challenges with the roll out of KM club in the beginning?**

*Prompt: Motivation (staff, kids), parent’s approval, access to facilities, weather, clothing, risk assessments, space, timetabling?*

- **What helped the most in setting up KM Club?**

*Prompts: External support, resources?*

*[Ask all teachers]*

- **How do you ensure the safety of children participating in KM Club?**

Prompts: Risk assessment, working with children check?

1. **Benefits, disadvantages and recommendations**

*[Ask all teachers]*

- **What do you believe are the benefits of KM Club?**

*Prompt: behaviour, health, engagement, attention?*

- **Do you believe there are any disadvantages to KM Club? If so, what are they?**

*Prompt:*  *Kids left out, supervision, tired in class*

- **Is there anything else you would like to add in regards to KM Club implementation?**
- **Finally, what top tips would you give to another school looking to start the KM Club?**

**End**

- Thank you
- Reiterate what will happen with the data and next steps (data recorded, transcribed, and combined with other interviews we are conducting and with student physical activity outcomes)
- Can I contact you again if I have any more questions?
